# Effect of Sodium Benzoate on Cognitive Function Among Patients With Behavioral and Psychological Symptoms of Dementia: Secondary Analysis of a Randomized Clinical Trial

**DOI:** 10.1001/jamanetworkopen.2021.6156

**Published:** 2021-04-21

**Authors:** Chieh-Hsin Lin, Ping-Kun Chen, Shi-Heng Wang, Hsien-Yuan Lane

**Affiliations:** 1 Department of Psychiatry, Kaohsiung Chang Gung Memorial Hospital, Chang Gung University College of Medicine, Kaohsiung, Taiwan; 2 Graduate Institute of Biomedical Sciences, China Medical University, Taichung, Taiwan; 3 School of Medicine, Chang Gung University, Taoyuan, Taiwan; 4 School of Medicine, China Medical University, Taichung, Taiwan; 5 Department of Neurology, China Medical University Hospital, Taichung, Taiwan; 6 Department of Occupational Safety and Health, China Medical University, Taichung, Taiwan; 7 Department of Psychiatry & Brain Disease Research Center, China Medical University Hospital, Taichung, Taiwan; 8 Department of Psychology, College of Medical and Health Sciences, Asia University, Taichung, Taiwan

## Abstract

**Importance:**

Female gender is a major risk factor for dementia; however, gender has not yet been adequately addressed by clinical trials. A recent study demonstrated that sodium benzoate, a D-amino acid oxidase inhibitor, improved cognitive function in early-phase Alzheimer disease.

**Objective:**

To examine the potential gender difference in the effects of benzoate treatment on the behavioral and psychological symptoms of dementia (BPSD).

**Design, Setting, and Participants:**

This post hoc secondary analysis used data from a randomized, double-masked, placebo-controlled trial conducted in 3 major medical centers in Taiwan and enrolled 97 patients with BPSD. Data were analyzed between February 2014 and November 2017.

**Interventions:**

Six weeks of treatment of 250 to 1500 mg/d of sodium benzoate or placebo.

**Main Outcomes and Measures:**

The primary outcome measures were Alzheimer disease assessment scale–cognitive subscale (ADAS-cog) and Behavioral Pathology in Alzheimer Disease Rating Scale (BEHAVE-AD) scores.

**Results:**

Among 97 total participants (62 [64%] women; mean [SD] age, 75.4 [7.7] years), 49 patients (30 women and 19 men) were randomized to sodium benzoate, and 48 (32 women and 16 men) were randomized to placebo. Among 62 women, 6-week benzoate treatment significantly surpassed placebo in the effects on ADAS-cog performance (mean [SD] difference in score between baseline and end point, −3.1 [6.4] points vs 0 [4.5] points; Cohen *d* = 0.56; *P* = .04) but not BEHAVE-AD performance. In contrast, among 35 men, the 2 treatment groups did not differ significantly in both ADAS-cog and BEHAVE-AD scores. Compared with placebo, benzoate treatment also increased estradiol to follicle-stimulating hormone ratios among women (mean [SD] difference between baseline and end point, 0 [0.2] vs −0.1 [0.3]; *P* = .03).

**Conclusions and Relevance:**

These findings suggest that benzoate treatment may improve cognitive function in women with later-phase dementia. In the future, longer dose-finding trials are warranted to further clarify the efficacy of benzoate for later-phase dementia and investigate the role of sex hormones and other factors in the pathogenesis of dementia.

**Trial Registration:**

ClinicalTrials.gov Identifier: NCT02103673

## Key Points

**Question:**

Is there a gender difference in the effectiveness of benzoate treatment for dementia with behavioral and psychological symptoms?

**Findings:**

In this post hoc secondary analysis of a randomized, double-masked, 6-week trial that included 97 patients with behavioral and psychological symptoms, benzoate significantly surpassed placebo in cognitive performance in women but not in men on 1 of 2 assessment measures. Benzoate also increased estradiol to follicle-stimulating hormone ratios among women.

**Meaning:**

These findings suggest that benzoate may improve cognitive function in women with later-phase dementia.

## Teaser

This secondary analysis of a randomized, 6-week clinical trial examines gender differences in the effectiveness of sodium benzoate in treating the behavioral and psychological symptoms of dementia.

## Introduction

Age and female gender are 2 major risk factors for Alzheimer disease (AD); two-thirds of older adults with AD are women. Even regarding the difference in longevity, studies suggest that women are still at a higher risk.^1^ However, gender has not yet been adequately addressed in clinical trials. More attention to gender differences will improve outcomes of older adults with dementia.^2^

Among various aging and dementia theories,^3^ altered N-Methyl-D-aspartate receptor (NMDAR)–related neurotransmission is involved in dementia manifestations, including cognitive and behavioral domains.^4,5^ NMDAR overactivation leads to neurotoxicity, while its hypofunction results in neurodegeneration,^6^ suggesting that NMDAR activity needs to be maintained at an optimal range.^7^ Behavioral and psychological symptoms of dementia (BPSD) develop in AD, vascular dementia (VaD), and other kinds of dementia.^8^ Depression is common in the early stage of dementia, while psychotic symptoms are more common in later stages.^9^ Behavioral and psychological symptoms are associated with worsening cognition, later phases of dementia, and poorer prognosis.^10^ In addition, studies revealed a transient serum glutamate elevation in acute phase of ischemia stroke and brain injury^11^ and consequently long-lasting NMDAR hypofunction.^12^ Glycine, an NMDAR co-agonist, was found to exert neuroprotective activity for ischemic stroke.^13^ Therefore, it is reasonable to treat dementia via NMDAR enhancement.^6,14^

One of the avenues to enhance NMDAR function is via inhibiting D-amino acids oxidase (DAAO) activity.^15^ In an earlier randomized, double-masked, placebo-controlled trial,^16^ sodium benzoate, a pivotal DAAO inhibitor,^17^ significantly improved the cognitive function of patients with early-phase AD (without BPSD), with a mean dose of 716.7 mg/d at week 24. On the other hand, in a 2019 randomized, double-blind, placebo-controlled trial on dementia with behavioral and psychological symptoms,^18^ benzoate did not surpass placebo in restoring cognitive function or reducing behavioral and psychological symptoms, while benzoate and placebo showed a similar safety profile.

A 1993 study^19^ showed that female rats were much more susceptible to NMDAR modulation than males. Another study found that the average density of NMDAR currents in dorsal root ganglia of female rats was 2.8-fold larger than that of male rats, and that addition of 17-β-estradiol increased NMDAR currents by 55% in the neurons of women, but only 19% in men, indicating sex differences in the activity and estrogen modulation of NMDAR.^20^ Levels of gonadotropins, such as follicle-stimulating hormone (FSH), are elevated in some patients with AD,^21^ and administration of sodium benzoate can lead to a significant decrease in the circulating levels of FSH compared with the control group in rats.^22^ Therefore, whether benzoate can benefit later-phase dementia in a sex-dependent manner deserves study. The current study, an exploratory secondary analysis of the previous work,^18^ aimed to examine whether benzoate can benefit the treatment for dementia with behavioral and psychological symptoms specifically in female patients.

## Methods

The clinical trial was approved by the institutional review boards of Kaohsiung Chang Gung Memorial Hospital, China Medical University Hospital, and Lin-Shin Hospital, and was registered with ClinicalTrials.gov (NCT02103673). The trial protocol and the statistical analysis have been previously published and are also available in Supplement 1.^18,23^ Written informed consent was obtained from all participants and guardians. Data analysis and reporting of this post hoc secondary analysis was done in accordance with the Consolidated Standards of Reporting Trials (CONSORT) reporting guideline.

The methods of this study have been described elsewhere.^18,23^ In summary, this was a randomized, double-masked, placebo-controlled, 6-week trial conducted by 3 major medical centers in Taiwan. Inclusion criteria included being aged between 50 and 100 years; probable AD (by National Institute of Neurological and Communicative Diseases and Stroke/Alzheimer’s Disease and Related Disorders Association criteria) or probable VaD (by National Institute of Neurological Disorders and Stroke and Association Internationale pour la Recherche et l’Enseignement en Neurosciences [NINDS-AIREN] criteria, and with a poststroke period ≥3 months); Mini-Mental State Examination (MMSE) scores of 5 to 26; Clinical Dementia Rating Scale score equal to or greater than 1; Behavioral Pathology in Alzheimer Disease Rating Scale (BEHAVE-AD) score equal to or greater than 2; 6 years or longer of education or work experience; and being capable of understanding the purpose, procedures, risks, and rights of the study.^18^ Exclusion criteria included history of substance dependence in the past 6 months or current substance abuse; other major psychiatric diagnoses such as schizophrenia, major depressive disorder, bipolar disorder, and intellectual disability; serious medical or neurological illness other than AD or VaD and other secondary dementia; and inability to follow the protocol.^18^

Patients were randomized through a computer-generated randomization table to receive active treatment or placebo in a 1:1 ratio. Medication was provided with identical-appearing capsules of sodium benzoate or placebo.

The primary outcomes included the Alzheimer disease assessment scale-cognitive subscale (ADAS-cog) assessed at baseline and end point (scored on a 70-point scale, with 70 indicating higher impairment), and BEHAVE-AD measured at weeks 0, 2, 4, and 6 (scored on a 75-point scale, with 75 indicating more pronounced AD symptoms). The secondary outcomes included the Neuropsychiatric Inventory, Instrumental Activities of Daily Living, Zarit Caregiver Burden Interview (measured at baseline and end point), and Geriatric Depression Scale at weeks 0, 2, 4, and 6.^18^

Under a medium effect (Cohen *f* = 0.33), the minimal sample size required was 74 to achieve a group difference ADAS-cog score of 4, with SD estimated at 6.^18^ Finally, 97 patients with BPSD were allocated to receive from 250 to 1500 mg/d of sodium benzoate or placebo. The dose was started at 250 to 500 mg/d, followed by a biweekly increment of 250 to 500 mg/d, if clinically indicated.

### Laboratory Measurements

#### Estradiol Assay

Concentrations of estradiol in plasma and FSH in serum were measured at baseline and end point of benzoate or placebo treatment. Estradiol protein concentrations were measured using commercially available enzyme-linked immunosorbent assay kits according to the manufacturer’s recommended protocol (DRG International). Briefly, 100 μL of plasma samples or the standard was added to each well of a 96-well plate, and 200 μL of enzyme-conjugate was then incubated for 4 hours at room temperature. The liquid was then removed. Each well was washed 3 times with wash buffer. Thereafter, 200 μL of substrate solution was added to each well and then incubated for 30 minutes at room temperature with protection from the light. Finally, 100 μL of stop solution was added to each well, and thorough mixing was ensured. The absorbance at 450 nm was assessed with the Benchmark Plus Microplate Reader (Bio-Rad), and the concentrations of estradiol in the samples were determined using a standard curve.

#### FSH Assay

FSH concentrations were measured using commercially available enzyme-linked immunosorbent assay kits according to the manufacturer’s recommended protocol (DRG International). Briefly, 25 μL serum samples or standard was added to each well of a 96-well plate, and 100 μL enzyme conjugate reagent was added into each well and incubated for 30 minutes at room temperature. After being washed for 5 times with deionized water, each well was added with 100 μL substrate solution and incubated for 10 minutes at room temperature with protection from the light. Finally, 50 μL of stop solution was added to each well, and thorough mixing was ensured. The absorbance at 450 nm was assessed with the Benchmark Plus Microplate Reader (Bio-Rad), and the concentrations of FSH in the samples were determined using a standard curve.

### Statistical Analysis

Our gender analysis was conducted post hoc. A Fisher exact test was used to compare differences of categorical variables and 2-sample *t* test (or Mann-Whitney U test if the distribution was not normal) for continuous variables (including some demographic characteristics, ADAS-cog, and laboratory measurements) between the 2 treatment groups, with men and women in separate groups, and sex differences were also examined. Mean changes from baseline in repeated-measure assessments (weeks 2, 4, and 6) for BEHAVE-AD were assessed using the generalized estimating equation method with treatment, visit, and treatment-visit interaction as covariates; baseline value was used as the reference among men and women, grouped separately. No imputation for the incomplete data was used for the generalized estimating equation analysis. The working correlation matrix was specified as first-order autoregressive, named AR.^1^

Therapeutic effect sizes (Cohen *d*) were used to determine the magnitude of improvement for the continuous variables resulting from benzoate treatment vs placebo. Multiple linear regression analyses were used to generate models estimating treatment response. A Fisher exact test was used to compare the between-group differences in the dropout rates. All data were analyzed by IBM SPSS Statistics version 22.0 (SPSS Inc). Significance was based on 2-tailed tests with *P* < .05. Data analysis was conducted from February 2014 to November 2017.

## Results

As described in previously published results from this study,^18,23^ 121 patients were screened, and 97 among them were eligible and randomized (mean [SD] age, 75.4 [7.7] years; 62 [64%] women). A total of 84 patients (86.6%) completed the 6-week clinical trial. The dropout rate of the benzoate group (6 patients [12.2%]) was similar to that of the placebo group (7 patients [14.6%]). The participant flow diagram, which has been previously published,^23^ is shown in eFigure in Supplement 2. Demographic data (including age, age at illness onset, education level, and body mass index [BMI; calculated as weight in kilograms divided by height in meters squared] at baseline) were similar between the benzoate group and the placebo group in both men women (Table 1). The mean (SD) benzoate doses at weeks 2, 4, and 6 were 341.8 (121.8), 528.4 (248.3), and 622.0 (340.6) mg/d, respectively.^18^

**Table 1.** Baseline Demographic and Clinical Characteristics of 2 Treatment Groups in Male and Female Patients

Characteristics	Men		Women	
	Sodium benzoate (n = 19)	Placebo (n = 16)	Sodium benzoate (n = 30)	Placebo (n = 32)
Age, y	75.8 (5.7)	74.3 (7.2)	75.6 (7.9)	75.7 (9.1)
Age at onset, y	74.5 (6.3)	70.6 (7.4)	73.9 (7.7)	73.7 (9.5)
Education, y	5.9 (3.6)	6.6 (3.7)	3.8 (4.0)	3.8 (4.1)
BMI	22.6 (3.6)	24.7 (4.5)	22.7 (3.4)	23.9 (4.5)
CDR at baseline, No. (%)				
1	11 (57.9)	10 (62.5)	17 (56.7)	20 (62.5)
2	7 (36.8)	2 (12.5)	9 (30.0)	10 (31.3)
3	1 (5.3)	4 (25.0)	4 (13.3)	2 (6.3)

Abbreviations: BMI, body mass index (calculated as weight in kilograms divided by height in meters squared); CDR, Clinical Dementia Rating Scale.

### Cognitive Improvement With Benzoate Among Women

Among women, benzoate treatment (30 women) generated significantly greater improvement in the ADAS-cog score than placebo (32 women) (mean [SD] difference from baseline to end point, −3.1 [6.4] points vs 0 [4.5] points; Cohen *d* = 0.56, *t* = −2.09, *P* = .04) (Table 2).

**Table 2.** Results of Measures of ADAS-cog in Male and Female Patients Over the 6-Week Treatment

ADAS-cog measurement	Men							Women							*P* value for sex difference^b^
	Sodium benzoate		Placebo		Cohen *d*	*t*	*P* value^a^	Sodium benzoate		Placebo		Cohen *d*	*t*	*P* value^a^	
	Score, mean (SD)	Patients, No.	Score, mean (SD)	Patients, No.				Score, mean (SD)	Patients, No.	Score, mean (SD)	Patients, No.				
Baseline	29.9 (14.0)	19	34.5 (14.8)	15	NA	−0.92	.36	30.6 (11.8)	29	25.5 (10.5)	32	NA	1.79	.08	.07
End point	29.0 (13.7)	18	25.4 (15.5)	11	NA	0.65	.52	27.0 (13.3)	27	26.0 (11.8)	30	NA	0.29	.77	.67
Difference^c^	0.5 (7.4)	18	−5.1 (7.5)	11	.75	1.96	.06	−3.1 (6.4)	27	0 (4.5)	30	.56	−2.09	.04	.004

Abbreviations: ADAS-cog, Alzheimer Disease Assessment Scale-Cognitive Subscale; NA, not applicable.

^a^ Independent *t* test.

^b^ *P* value for interaction term between sex and treatment in regression analyses.

^c^ Baseline measurements of patients without end point data were excluded in calculating the difference.

 By contrast, among men, benzoate treatment (19 men) did not differ significantly from placebo (16 men) in altering ADAS-cog scores (mean [SD] difference from baseline to end point, 0.5 [7.4] points vs −5.1 [7.5] points; Cohen *d* = 0.75, *t* = 1.96, *P* = .06) (Table 2). There was a statistically significant sex difference in benzoate treatment on altering ADAS-cog scores (*P* = .004) (Table 2). In addition, there was no difference between benzoate and placebo groups in BEHAVE-AD scores after 6 weeks of treatment for the male (*z* = −0.05, *P* = .96) or female patients (*z* = 0.18, *P* = .86) (eTable in Supplement 2).

### Blood Levels of Hormones

Laboratory parameters were measured at baseline and end point. Among women, benzoate treatment increased estradiol to FSH ratios significantly from baseline to end point compared with placebo (mean [SD] difference, 0 [0.2] vs −0.1 [0.3]; *P* = .03), while FSH levels and estradiol levels did not change significantly (Table 3). Changes of FSH levels, estradiol levels, and estradiol to FSH ratios did not differ significantly between benzoate and placebo groups, both among all participants and men, grouped separately (data not shown).

**Table 3.** Measures of Hormones Over the 6-Week Treatment Between Sodium Benzoate and Placebo Groups in Female Patients

Measures	Sodium benzoate		Placebo		*P* value
	Mean (SD)	Participants, No.	Mean (SD)	Participants, No.	
**Estradiol, pg/mL**					
Baseline	25.7 (18.9)	30	24.1 (17.6)	31	.81^a^
End point	26.3 (16.7)	29	23.5 (16.9)	27	.33^a^
Difference	0 (5.6)	29	−1.1 (5.9)	27	.45^a^
**FSH level, mIU/mL**					
Baseline	49.8 (16.3)	30	45.8 (18.8)	31	.37^b^
End point	48.0 (15.8)	29	45.9 (19.2)	27	.66^b^
Difference	−1.6 (9.1)	29	1.4 (7.7)	27	.19^b^
**Estradiol to FSH ratio**					
Baseline	0.6 (0.5)	30	0.7 (1.0)	31	.83^a^
End point	0.6 (0.5)	29	0.7 (0.8)	27	.79^a^
Difference	0 (0.2)	29	−0.1 (0.3)	27	.03^a^

Abbreviation: FSH, follicle-stimulating hormone.

SI conversion factors: To convert estradiol to pmol/L, multiply by 3.671; FSH to IU/L, multiply by 1.0.

^a^ Mann-Whitney *U* test for Shapiro-Wilk normality test <.05.

^b^ Independent *t* test.

## Discussion

It is important but difficult to treat patients with later-phase dementia, especially those with behavioral and psychological symptoms.^8,24^ Our previous study demonstrated that sodium benzoate, an indirect NMDAR enhancer, benefited cognitive function of both men and women with early-phase dementia.^16^ The current study suggests that benzoate may improve cognitive function of women (but not men) with later-phase dementia with behavioral and psychological symptoms, therefore lending support to the previous notion that women may be more susceptible to NMDAR modulation than males, which was based upon animal study.^19^

Psychosis leads to faster cognitive decline in patients with dementia.^25^ Though antipsychotics have been widely used for the treatment of dementia-related psychosis,^26,27^ no medications have been approved for the treatment.^28^ Benzoate exerted antipsychotic properties in a mouse model^29^ and in patients with chronic schizophrenia^30,31^; however, it was unable to reduce the psychotic symptoms in patients with dementia in the current study. One of the possible reasons is that the dosage (341.8 ± 121.8 mg/d at week 2, 528.4 ± 248.3 mg/d at week 4, and 622.0 ± 340.6 mg/d at week 6) of the current study appears much lower than that (1000 or 2000 mg/d) used in the studies on schizophrenia.^30,31^ Future studies that examine the effects of higher doses are needed.

In the current study, benzoate treatment improved cognitive function significantly among women according to the ADAS-cog measure (Table 2) and increased estradiol to FSH ratios compared with placebo (Table 3). The findings were in accordance with that from the previous study, which showed that the administration of sodium benzoate decreased the circulating levels of FSH in rats.^22^ Sex hormones were found to modulate hippocampal NMDAR expression in mice^32^ and interact with circulating antioxidants in human blood.^33^ Of note, benzoic acid ester of estrone, a precursor of estradiol, showed prolonged duration of action^34^; moreover, p-hydroxybenzoic acid, a phenolic derivative of benzoic acid, and other alkyl hydroxy benzoate preservatives displayed estrogenic effects from in vitro and in vivo studies.^35,36^ Of note, sodium benzoate treatment did not significantly change estradiol or FSH, and the minor differences in the ratio was too small to have any clinical value. Further study is needed to explore other possible mechanisms in benzoate effects on dementia or other brain disorders.^37^ On the other hand, dementia treatment usually takes time much longer than 6 weeks.^38^ While long-term use of estrogen replacement has been found to be associated with an increased risk of AD in older adults,^39^ the benefit vs risk of long-term benzoate treatment deserves further studies.

Oxidase stress has been implicated in pathogenesis of dementia. Importantly, gender difference has been found in oxidative metabolism, with decreased production of cyclooxygenase metabolites in neutrophils from women, which in part derives from an increased oxidative burst activity.^40^ Sodium benzoate also plays a role as an intracellular oxygen-centered radical scavenger for the hydroxyl radical.^40,41^ Whether sodium benzoate exerts its action in the treatment of dementia via this mechanism deserves further study as well.

In our previous paper,^23^ after benzoate treatment, DAAO inhibition was correlated with ADAS-cog decrease. However, the change of DAAO was similar between male and female patients (*t* = 0.69, *P* = .50). On the other hand, whether there is gender difference in benzoate’s pharmacokinetics has been unclear.^42,43^ More studies on pharmacodynamics and pharmacokinetics of benzoate in men and women are needed in the future.

Sodium benzoate has antibacterial and antifungal activity.^44^ Accumulating evidence shows the role of brain-gut-microbiota axis in neuropsychiatric disorders.^45,46^ It is of interest to investigate the role of gut microbiota before and after treatment of benzoate in the future.

In previous studies, results showing benzoate’s efficacy for schizophrenia or early psychosis has been diverse^30,31,47,48^: it reduced psychotic symptoms of patients with chronic schizophrenia in 2 studies,^30,31^ but not in another study conducted in Taiwan^47^; it also didn’t decrease symptoms in patients with early psychosis in a study set in Australia.^48^ Whether the findings of the current study can be extrapolated to other populations with different ethnicity, illness course, or treatment history requires further studies.

### Limitations

This post hoc secondary analysis of a randomized clinical trial had several limitations. First, our findings are limited by the short duration of treatment. The usual course of treatment needs 6 to 12 months to ameliorate the cognitive decline of dementia.^38^ In comparison with the 24-week benzoate treatment for early-phase dementia,^16^ the current 6-week trial may have been too short to achieve the full response. Second, the average dose of benzoate in this study is lower than that of the previous study on early-phase dementia (716.7 mg/d)^16^ and the study on schizophrenia (1000 or 2000 mg/d at week 6).^31^ Third, the sample size in this study was limited and there were more women than men in the sample, which posed a power issue in detecting effects in men and women regarding sex differences. We calculated the statistical power by using GPower software. On the basis of the current scenario with 62 female participants, effect size of 0.56, and α = .05, the power was 0.70 in a 1-sided test. However, the statistical power in men (with 35 participants) was inadequate. Further studies with larger sample sizes are needed to validate the exploratory analysis. Fourth, because of the small sample size, the large differences of the ADAS-cog scores at baseline in men and women were not statistically significant (Table 2). Future studies are required to get a balanced distribution at baseline to reduce confounding and bias. Fifth, multiple hypothesis tests were conducted to assess various associations between benzoate treatment and several outcomes in male and female patients. Hence the chances of false-positive findings may have been increased. Our results thus should be considered exploratory.

## Conclusions

When considering the results of this post hoc secondary analysis of a randomized clinical trial on later-phase dementia with behavioral and psychological symptoms alongside our previous clinical trial^16^ on early-phase dementia, sodium benzoate, an indirect NMDAR agonist, can improve the cognitive function of all patients with early-phase dementia, but only benefit the cognitive function of female patients with later-phase dementia and behavioral and psychological symptoms. More studies are warranted to elucidate the underlying mechanisms. Notably, for women with later-phase dementia and behavioral and psychological symptoms, a short-term (only 6-week) treatment of benzoate may improve cognitive function, while for patients with early-phase dementia, 16 weeks of benzoate treatment is required to benefit cognitive function.^16^

Diverse manifestations and courses of dementia may represent numerous pathogeneses. Whether sodium benzoate can help at least a portion of dementia patients, such as women or patients at a younger age and earlier phase of illness, deserves more studies with longer duration and higher doses (and perhaps also lower doses) for confirmation.

## Notes

Supplement 1. **Trial Protocol**

Supplement 2. **eTable.** Measures of BEHAVE-AD Over the 6-week Treatment Using Generalized Estimating Equations (GEE) Method **eFigure.** Participant Flow Diagram

Supplement 3. **Data Sharing Statement**
