# Supplementary material for: Effect of Sodium Benzoate on Cognitive Function Among Patients With Behavioral and Psychological Symptoms of Dementia: Secondary Analysis of a Randomized Clinical Trial
Source: JAMA Netw Open. 2021 Apr 21;4(4):e216156. doi: 10.1001/jamanetworkopen.2021.6156 (PMC8060832; doi:10.1001/jamanetworkopen.2021.6156)
Supplement: Supplement 1. — Trial Protocol [file jamanetwopen-e216156-s001.pdf]

Clinical Trial Protocol: Sodium Benzoate for the Treatment of Cognitive Function and Behavioral and Psychological Symptoms of Dementia

Background: The increasing prevalence of dementia in the elderly is a heavy burden of both patients and their family. Behavioral and Psychological Symptoms of Dementia (BPSD), which is particularly disturbing to caregivers, needs further investigation for its etiology and associative factors. The current main hypothesis for Alzheimer’s disease (AD) is the deposit of β–amyloid in the brain. However, interventions aiming to the clearance of β–amyloid deposit all failed to improve the cognitive or global function of AD to date, implying that there should be other more important mechanisms unproven in the course of AD.

NMDA receptor (NMDAR) activation plays an important role in learning and memory. NMDARs were found to decrease in the frontal lobe and hippocampus of patients with AD. Our previous study found that NMDAR enhancer-sodium benzoate (a D-amino acid oxidase inhibitor which can elevate D-serine level) can significantly improve the cognitive function of patients with early-phase AD, and also benefit a portion of moderate to severe AD patients. Therefore, we plan to test whether sodium benzoate can benefit patients with BPSD.

Methods: We plan to enroll patients with BPSD. The following tools will be used for the assessments of clinical and cognitive function: Clinical Dementia Rating (CDR), Mini Mental Status Examination (MMSE), Behavioral Pathology in Alzheimer′ s Disease Rating Scale (BEHAVE-AD), Alzheimer's disease assessment scale-cognitive subscale (ADAS-cog).

Patients will be recruited from Kaohsiung Chang Gung Memorial Hospital, China Medical University Hospital, and Lin-Shin Hospital, which are 3 major medical centers in Taiwan. The study has been approved by the Institutional Review Boards of the aforementioned hospitals, and will be conducted in accordance with the current revision of the Declaration of Helsinki.

Patients will be evaluated by research psychiatrists after a thorough medical and neurological workup. Written informed consent will be obtained from all participants or guardians of participants in this study.

Subjects will be randomly assigned into two treatment groups in a double-blind manner: (1) sodium benzoate (initial dose: 250–500 mg/day, dose range: 250–1500 mg/day), (2) placebo, for 6 weeks. For patients who received acetylcholine esterase inhibitors (AChEIs) before entering the trial, AChEIs need to be maintained at an optimal and stable dose for at least 6-week prior to the randomization period of this study, and the AChEI regimens will remain unchanged during the period of this NMDAR enhancer trial. For patients who used antipsychotics before entering the trial, antipsychotics need to be maintained at an optimal and stable dose for at least 3-week prior to this study and the dosage will remain unchanged during the period of this trial.

The ratio of patient with diagnosis of Alzheimer’s disease / vascular dementia and patients with / without AChEI treatment is expected to be similar in both groups.

During the trial, the dosage of each compound will increase every 2 weeks (14 days) according to the result of clinical assessment and if patients are tolerant. The dose of benzoate will be titrated by 250-500 mg/day every 2 weeks, if it is clinically necessary. We will measure their psychiatric and behavioral symptoms at Week 0, 2, 4, 6 and evaluate patient’s cognitive function before and after the trial.

To ensure concealment of the randomization assignment, we will prepare two kinds of capsules, one containing sodium benzoate and the other containing placebo. Sodium benzoate (250 mg) and identical-appearing placebo will be purchased from qualified pharmaceutical company. Drug compliance will be ensured by care-givers.

Patients will be randomized through a randomization table to receive placebo or any of active treatments in a 1:1 ratio.

During the study, limited use of benzodiazepines (for example, up to 4-mg/day lorazepam) is allowed as concomitant medication for agitation or insomnia. Except the subjects' concomitant CNS medication that has been taken before and during the trial, no other centrally acting drugs are permitted.

In case of side-effect intolerance or clinical worsening, the patients will be withdrawn earlier.

Includion and exclusion criteria

Patients will be enrolled if they [1] satisfy NINCDS-ADRDA (National Institute of Neurological and Communicative Disorders and Stroke and the Alzheimer's Disease and Related Disorders Association) (McKhann et al., 1984) criteria for probable AD, or criteria for probable VaD from the National Institute of Neurological Disorders and Stroke and Association Internationale pour la Recherche et l'Enseignement en Neurosciences (NINDS-AIREN) (Roman et al., 1993); [2] are 50 or over of age; [3] for patients with VaD, have the post-stroke period of 3 months or more; [4] have Mini-Mental State (MMSE) scores of 5-26; [5] have severity scores of Clinical Dementia Rating (CDR) (Morris, 1993) of 1 or higher; [6] have Behavioral Pathology in Alzheimer's Disease Rating Scale (BEHAVE-AD) (Reisberg et al., 1987) score 2 or higher, and also have delusions, hallucinations, aggression, agitation, or other behavioral disturbances that developed after the onset of dementia and are severe enough to disrupt their functioning; [7] are literate with ⩾6 years of education or with working experiences; and [8] are able to understand the purpose, flow, risks and rights of the study, and willing to sign the informed consent form.

Exclusion criteria includes [1] current substance abuse or history of substance dependence in the past 6 months; [2] other major psychiatric diagnoses, such as schizophrenia, major depressive disorder, bipolar disorder and mental retardation etc.; [3] serious medical or neurological illness other than AD, VaD, and other dementia; and [4] inability to follow protocol.

Assessments

Measures of efficacy

The primary outcome measure is change of Alzheimer's disease assessment scale-cognitive subscale (ADAS-cog) score and BPSD symptoms measured by total scores of BEHAVE-AD (Reisberg et al., 1987).

Secondary outcome measures include changes in scores of Neuropsychiatric Inventory (NPI) (Cummings, 1997), Instrumental Activities of Daily Living (IADL)(Lawton and Brody, 1969), Zarit Caregiver Burden Interview (J-ZBI)(Zarit et al, 1980; Ko et al, 2008) for caregivers and Geriatric Depression Scale (GDS)(Sheikh, 1986).

All assessments will be completed by well-trained psychometricians who are blind to treatment assignment. Each subject should have a caregiver who can ensure medication compliance. At each visit, both the patient and caregiver will be systematically asked for any side effect. Physical and neurological examinations will be performed at each visit. Patients may be receiving concomitant lorazepam.

Measures of safety

Systemic side effects are reviewed by applying the Udvalg for Kliniske Undersogelser (UKU) Side-effects Rating Scale (Lingjaerde, 1987). These side effect assessments are also performed biweekly at each visit. Blood routine and blood biochemistry will be conducted at weeks 0 and 6.

Clinical ratings will be performed by the research psychiatrists and neurologists who are trained and experienced in the rating scales. Inter-rater reliability will be analyzed with the ANOVA test. Only raters reaching the intra-class correlation coefficients of 0.90 during pre-study training will be allowed to rate the patients. To minimize inter-rater variability, each individual patient will be assessed by the same rater throughout the trial.

Data Analysis

To ensure a sufficient sample size, a power analysis will be conducted (Faul et al., 2009) and a power of 80% will be obtained. Under the assumption of the medium effect (Cohen's f = 0.33), the sample size required per group will be 37 to achieve a group-difference of ADAS-cog score at 4, with S.D. estimated at 6. For further confirmation, we will increase the sample size per group to about 45, which will be larger than that (n = 30) of the previous benzoate trial in early-phase AD (Lin et al., 2014a).

Chi-square test (or Fisher's exact test) will be used to compare differences of categorical variables and Student's two-sample t-test (or Mann-Whitney U test if the distribution will not be normal) for continuous variables (including some demographic characteristics, ADAS-cog, and laboratory measurements) between two groups. Mean changes from baseline in repeated-measure assessments (weeks 2, 4, and 6) (BEHAVE-AD and GDS) will be assessed using the generalized estimating equation (GEE) method with treatment, visit and treatment-visit interaction as fixed effects and intercept as the only random effect; baseline value as the covariance. No imputation for the incomplete data will be used for the GEE analysis. The working correlation matrix will be specified as autoregressive of order 1, named AR(1).

Multiple linear regression analyses will be used to generate predictive models for treatment response.

Fisher's exact test will be used to compare the between-group differences in the dropout rates. All data will be analyzed by IBM SPSS Statistics (version 22.0; SPSS Inc.). All p values will be based on two-tailed tests with a significance level of 0.05.

Expected results: We hypothesize that sodium benzoate may yield better efficacy than placebo for cognitive function and clinical symptoms in patients with BPSD. We predict that sodium benzoate will benefit patients with BPSD with favorable safety profile and tolerance.
