# Supplementary material for: Effect of Sodium Benzoate on Cognitive Function Among Patients With Behavioral and Psychological Symptoms of Dementia: Secondary Analysis of a Randomized Clinical Trial
Source: JAMA Netw Open. 2021 Apr 21;4(4):e216156. doi: 10.1001/jamanetworkopen.2021.6156 (PMC8060832; doi:10.1001/jamanetworkopen.2021.6156)
Supplement: Supplement 2. — eTable. Measures of BEHAVE-AD Over the 6-week Treatment Using Generalized Estimating Equations (GEE) Method eFigure. Participant Flow Diagram [file jamanetwopen-e216156-s002.pdf]

Supplemental Online Content

Lin CH, Chen PK, Wang SH, Lane HY. Effect of sodium benzoate on cognitive function among patients with behavioral and psychological symptoms of dementia: secondary analysis of a randomized clinical trial. *JAMA Netw Open*. 2021;4(4):e216156. doi:10.1001/jamanetworkopen.2021.6156

eTable. Measures of BEHAVE-AD Over the 6-week Treatment Using Generalized Estimating Equations (GEE) Method
eFigure. Participant Flow Diagram

This supplemental material has been provided by the authors to give readers additional information about their work.

© 2021 Lin CH et al. *JAMA Network Open.*

eTable 1. Measures of BEHAVE-AD Over the 6-week Treatment Using Generalized Estimating Equations (GEE) Method

	**Sodium benzoate Mean ± SD (*n*)**	**Placebo Mean ± SD (*n*)**	**Estimate***	**S.E.**	***Z***	***P* Value**
Male						
Baseline	9.9 ± 7.7 (19)	12.1 ± 11.4 (16)				
Week 2	6.7 ± 5.4 (19)	9.9 ± 9.6 (16)	-2.2613	1.6116	-1.40	.16
Week 4	6.3 ± 4.1 (18)	6.9 ± 4.4 (13)	-3.0315	1.4326	-2.12	.03
Week 6	5.5 ± 4.0 (17)	5.2 ± 3.6 (12)	-4.4082	2.0246	-2.18	.03
Endpoint	5.9 ± 4.2 (19)	7.9 ± 9.0 (16)	-3.8801	1.9549	-1.98	.05
Drug			-1.7591	3.0768	-0.57	.57
Week 2 x drug			-1.0261	1.9078	-0.54	.59
Week 4 x drug			-0.2501	1.8403	-0.14	.89
Week 6 x drug			0.3176	2.4168	0.13	.90
Endpoint x drug			-0.1185	2.3385	-0.05	.96
Female						
Baseline	11.3 ± 8.2 (30)	10.8 ± 7.8 (32)				
Week 2	10.0 ± 8.8 (30)	9.5 ± 6.5 (31)	-1.4534	0.6349	-2.29	.02
Week 4	10.2 ± 8.2 (25)	6.9 ± 4.7 (28)	-3.5276	0.9680	-3.64	<.001
Week 6	8.1 ± 6.8 (24)	8.3 ± 6.8 (29)	-1.9142	0.9751	-1.96	.05
Endpoint	9.6 ± 8.9 (30)	8.9 ± 7.1 (32)	-1.8629	0.9149	-2.04	.04
Drug			0.4391	1.9596	0.22	.82
Week 2 x drug			0.0948	1.1502	0.08	.93
Week 4 x drug			3.0225	1.4650	2.06	.04
Week 6 x drug			0.1928	1.5394	0.13	.90
Endpoint x drug			0.2565	1.4637	0.18	.86

* Estimate is the coefficient of treatment-visit interaction term in the GEE method with treatment, visit and treatment-visit interaction as covariates; baseline value as the reference. No imputation for the incomplete data was used. An autoregressive AR(1) covariance matrix was fit to the within-patient repeated measures. P values were based on two-tailed tests.
In the "Estimate" column for BEHAVE-AD, the upper 4 rows are for the placebo group only, while the bottom 5 are for benzoate, placebo interaction terms.
Abbreviations: BEHAVE-AD, Behavioral Pathology in Alzheimer's Disease Rating Scale.

© 2021 Lin CH et al. *JAMA Network Open.*

eFigure 1. Participant Flow Diagram

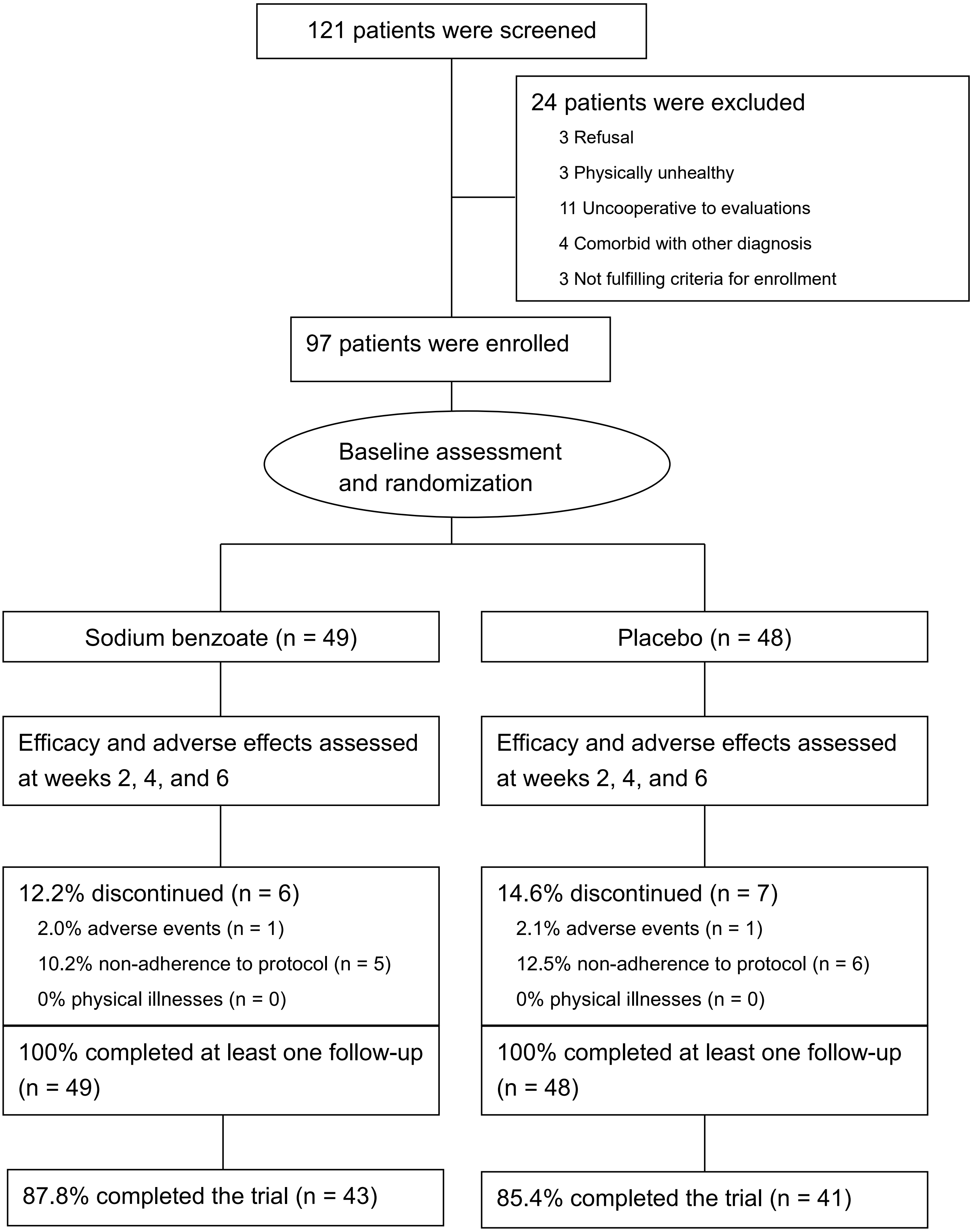

© 2021 Lin CH et al. *JAMA Network Open.*
