# Supplementary material for: Effect of Sodium Benzoate on Cognitive Function Among Patients With Behavioral and Psychological Symptoms of Dementia: Secondary Analysis of a Randomized Clinical Trial
Source: JAMA Netw Open. 2021 Apr 21;4(4):e216156. doi: 10.1001/jamanetworkopen.2021.6156 (PMC8060832; doi:10.1001/jamanetworkopen.2021.6156)
Supplement: Supplement 3. — Data Sharing Statement [file jamanetwopen-e216156-s003.pdf]

Lin C-H, Chen P-K, Wang S-H, Lane H-Y. Effect of sodium benzoate on cognitive function among patients with behavioral and psychological symptoms of dementia: secondary analysis of a randomized clinical trial. *JAMA Netw Open*. 2021;4(4):e216156. doi:10.1001/jamanetworkopen.2021.6156

Data Sharing Statement

Data

Data available: No

Additional Information

Explanation for why data not available: The data will be available under request approved by IRB.
